# P50 - A new modality using breath sound analysis in pediatric asthma

**DOI:** 10.1186/2045-7022-4-S1-P105

**Published:** 2014-02-28

**Authors:** Evgenii Furman, Elena Yakovleva, Sergey Malinin, Gregory Furman, Victor Meerovich, Vladimir Sokolovsky

**Affiliations:** 1 Perm State Medical Academy, Perm, Russia; 2 Ben-Gurion University of the Negev, Beer-Sheva, Israel

## 

Breath sound analysis is a useful tool in diagnostics of airway obstruction in pediatric respiratory organs’ pathology. To examine the breath sound various methods of frequency analysis of sound signalsare applied, include Wavelet and Fast Fourier Transform.

To develop more sensitive and stable diagnostic technique we proposed a new approach for computing analyzing of pediatric breath sounds: two-dimension frequency representation. This approach is based on the two-dimension Fast Fourier Transform allows one to compare and simultaneously analyze signals from two sound sensors. This method was applied in diagnostic of pediatric asthma.

The breath sound was measured by three sensors located on the right anterior chest, trachea, and inside mouth and acquired by a computer. Signals of various combinations of two sensors were analyzed. Children were considered eligible and included in research if their parents or guardians signed written informed consent. Numerical analyses are performed using the developed software based on the MATLAB package. The proposed method was applied in analyses of the sound signals of five patients. The patients’ signals were compared with signals of five healthy children. It was shown that the proposed two-dimension spectroscopy decreases noise background and more clearly shows the spectrum disturbances caused by the diseases than the spectrum analyses of the signal of a single sensor. The developed method is less sensitive to the sensor location.

